# Client Perceptions of Quality and Choice at Static, Mobile Outreach, and Special Family Planning Day Services in 3 African Countries

**DOI:** 10.9745/GHSP-D-18-00047

**Published:** 2018-10-03

**Authors:** Leah Jarvis, Jane Wickstrom, Caitlin Shannon

**Affiliations:** a EngenderHealth, New York, NY, USA. Now with Population Council, New York, NY, USA.; b EngenderHealth, New York, NY, USA. Now with the Bill & Melinda Gates Foundation, Seattle, WA, USA.; c EngenderHealth, New York, NY, USA.

## Abstract

**Background:**

Use of long-acting reversible contraceptives (LARCs) has grown rapidly in the Democratic Republic of the Congo (DRC), Tanzania, and Uganda. Uptake of LARCs is particularly high during mobile outreach and special family planning day events. It is therefore important to examine client perceptions of and experiences with full, free, and informed choice (FFIC) in different service delivery modalities.

**Methods:**

Between April and July 2015, we conducted a cross-sectional family planning client survey to assess FFIC and client satisfaction at static, mobile outreach, and special family planning day services in the DRC (n=9 sites), Tanzania (n=13), and Uganda (n=8). The study investigated clients' perceptions across 13 elements of FFIC, including measures of the quality of counseling and respondent satisfaction with services across the service delivery approaches. Composite FFIC scores were constructed and analyzed as the proportion of women who reported affirmatively to all elements and the mean score of positive responses. Satisfaction was assessed using a 4-point Likert scale. We used logistic regression to assess the association between the primary outcomes and mode of service delivery.

**Results:**

In total, we interviewed 585 women (n=150 in Uganda, n=200 in Tanzania, and n=235 in the DRC). The large majority of clients in all countries and modalities received their method of choice. Clients of mobile outreach and special family planning days preferred LARCs and permanent methods, particularly implants, compared with clients at static services. Composite measures of FFIC were lower for mobile outreach than for static services in Tanzania among all family planning clients (odds ratio [OR]=0.5; *P*≤.001) and among LARC clients specifically (OR=0.5; *P*≤.01); no significant differences were found in the DRC or Uganda. A mean FFIC score among all family planning clients showed that clients in all modalities in all countries reported experiencing most elements of FFIC, with averages ranging from 4.8 to 6.1 of 7 elements. Among LARC clients specifically, mean scores ranged from 8.3 to 9.8 of 11 elements. Where greater proportions of clients experienced higher FFIC, greater proportions of clients also tended to report being “very satisfied” with aspects of services and counseling.

**Conclusions:**

The results underscore that special family planning days and mobile outreach services are important and viable ways to increase women's access to family planning services, notably to LARCs, but further attention to respecting and fulfilling clients' full, free, and informed choice across all service delivery modalities is required.

## Abstract (precis)

In all 3 countries, nearly all women obtained their method of choice, with more mobile outreach and special family planning day clients having a preexisting preference for implants than static service clients. Clients of all service modalities in all countries reported experiencing most elements of full, free, and informed choice, but there is room for improvement with some aspects, such as counseling about potential side effects and giving clients the opportunity to ask questions.

Résumé en français à la fin de l'article.

## BACKGROUND

In many sub-Saharan African countries, service delivery modalities such as mobile outreach services and special family planning days play an important role in increasing the use of modern contraception, especially underutilized, long-acting reversible contraceptives (LARCs) and permanent methods.^1^^–^^7^

Mobile outreach services are crucial for increasing equitable access. By design, they serve poorer, marginalized, and geographically hard-to-reach communities and populations.^4^^,^^7^^–^^9^ Such services are characterized by the deployment of trained providers to lower-level health facilities or temporary set-ups—such as tents or community spaces—that are equipped with the required contraceptives and supplies.^6^^,^^7^^,^^9^^,^^10^

Special family planning days are distinct events that are well-advertised in the community and organized at higher-level facilities during which a full range of contraceptive methods—including LARCs and, often, permanent methods—are available. Trained providers and counselors assemble in sufficient numbers to dedicate themselves to family planning for the day and plan to have sufficient stock on hand, thereby creating confidence in the community and among clients that family planning methods will be available.

Both of these non-static service delivery modalities increase access to family planning, especially to LARCs, for women who may not have routine access to a wide contraceptive method mix or to family planning at all. Although short-acting methods, such as hormonal injections and pills, are highly popular with family planning adopters in sub-Saharan Africa, studies show that when women are able to choose among a wide range of contraceptive options, significant proportions choose LARCs.^1^^,^^5^^,^^9^^,^^11^^,^^12^ Due in part to global investments since the 2012 London Summit for Family Planning, hormonal implants have become more available and, as a result, women are especially likely to select implants, as compared with other contraceptive methods, including intrauterine devices (IUDs), when they are available.^5^^,^^8^^,^^13^^–^^16^ Although implants continue to represent a smaller proportion of the method mix in sub-Saharan Africa, they are one of the most rapidly growing contraceptive methods globally.^12^

Non-static service delivery modalities, such as mobile outreach and special family planning days, increase access to family planning, especially to LARCs.

EngenderHealth's Expand Family Planning (ExpandFP) Project (2013–2018), funded by the Bill & Melinda Gates Foundation, aimed to increase contraceptive options, with a focus on LARCs, for women and girls in the Democratic Republic of the Congo (DRC), Tanzania, and Uganda. The project began shortly after the 3 countries set aggressive goals at the London Summit: the government of DRC committed to achieving a national contraceptive prevalence rate (CPR) of 19% by 2020; Uganda's government committed to reducing unmet need to 10% by 2022; and Tanzania committed to doubling the number of family planning users to reach a national CPR of 60% by 2015.^17^ Although Tanzania did not achieve 60% CPR within their stated time frame, the government recommitted to its family planning program, adding financial resources and a pledge to increase the availability of youth-friendly health services.^18^ All 3 countries recommitted to their family planning goals at the London Summit meeting in July 2017, adding pledges to protect, respect, and fulfill client rights to full, free, and informed contraceptive choice.^19^

Following the 2012 London Summit, DRC, Tanzania, and Uganda set ambitious goals to increase the CPR and reduce unmet family planning need.

As a result of public- and private-sector family planning initiatives supported by governments, donors, and technical assistance partners, significant progress has been made in family planning use overall, and in use of LARCs more specifically. For example, in Kinshasa, DRC, rates of LARC/permanent method adoption increased quickly—from 2.5% prevalence in 2013 to 5.3% in 2015—as these methods became more available.^20^ In Uganda, the 2016 Demographic and Health Survey (DHS) showed that the overall CPR was increasing, with the prevalence of implant use among married women more than doubling in 5 years—from 2.7% in 2011 to 6.3% in 2016—and with IUDs remaining a much smaller proportion of the method mix, but still tripling from 0.5% to 1.5%.^21^^,^^22^ In Tanzania, the prevalence of implants nearly tripled among married women between the 2010 and 2015 DHS—from 2.3% to 6.7%—though IUDs remained below 1.0%.^23^^,^^24^

In all 3 countries, EngenderHealth's program introduced a voluntary, human rights-based approach and framework at national and project implementation levels. The goal was to build provider awareness and capacity in voluntary family planning programming, including counseling, to ensure that clients were able to make full, free, and informed choices (FFIC) and that programs assured equity and quality in the provision of care.^25^^,^^26^

Full choice is defined as access to the widest range of methods possible. To what degree that is possible may depend on what methods are approved for use at the national level and any constraints on the type of facility or cadre of provider. Free choice is a voluntary decision, without barriers or coercion, about whether to use family planning and, if so, which method to use. Informed choice is a decision based on complete, accurate, and unbiased information about family planning method options, including benefits, side effects, risks, and information about the correct use of the method chosen and the risks of family planning nonuse.^27^ The concepts of quality counseling and FFIC are interrelated and fall within a larger framework of client rights. The FFIC framework includes many elements of counseling quality, such as information on the array of contraceptive options available to the client and the benefits and side effects of each option, and how to discontinue use, when the client wants or needs to have an IUD or implant removed. However, FFIC goes beyond counseling quality and other measurement frameworks by assessing a client's ability to obtain her method of choice and by asking her who is primarily responsible for deciding whether to use family planning and which method. These specific elements of FFIC are influenced by factors that are external to provider counseling, such as method availability, cost of methods, and spousal influence.

To enhance FFIC, the ExpandFP Project invested resources into focusing on family planning clinical and counseling training for providers, facilitative supervision, infrastructure and contraceptive security improvements, community engagement, and advocacy. The project supported national trainers to train family planning providers on clinical contraceptive methods and rights-based counseling techniques. Trainings were followed by onsite provider follow up and coaching. Facilitative supervision was conducted at least twice a year in higher-level supported facilities where special family planning days occurred. The interventions supported service delivery for both short-acting (pills, injectables, and condoms) and long-acting methods (implants and IUDs). Permanent methods—male and female sterilization—were not offered at study facilities in the DRC but were available at most facilities in Tanzania and Uganda.

Project-supported public-sector special family planning days used community health workers, mass media, and dedicated family planning providers. In addition, the project-supported public-sector mobile outreach teams of dedicated family planning providers to serve lower-level facilities in remote areas with short-acting, long-acting, and sometimes permanent family planning methods. For both special family planning days and mobile outreach, community mobilizers were engaged to inform the community about upcoming events. Although special family planning days were conducted at facilities that generally had the most methods available, they frequently struggled with stock-outs and provider unavailability, while mobile outreach events typically occurred in smaller facilities that usually only had short-acting methods. In Tanzania and Uganda, all family planning methods were provided free to clients at public facilities at all times. In the DRC, family planning methods were only free to clients during special family planning days and at mobile outreach events. Special family planning days and mobile outreach events served higher client loads than routine services in all 3 countries and were particularly high in the DRC and Tanzania. On average, for special family planning days, 97 clients were served per day in the DRC, 112 in Tanzania, and 33 in Uganda. On average, during mobile outreach events, an average of 60 clients were served per day in the DRC, 133 in Tanzania, and 23 in Uganda. During these events, clients obtained information both from group education/counseling sessions and from individual counseling with the provider.

**Figure:**
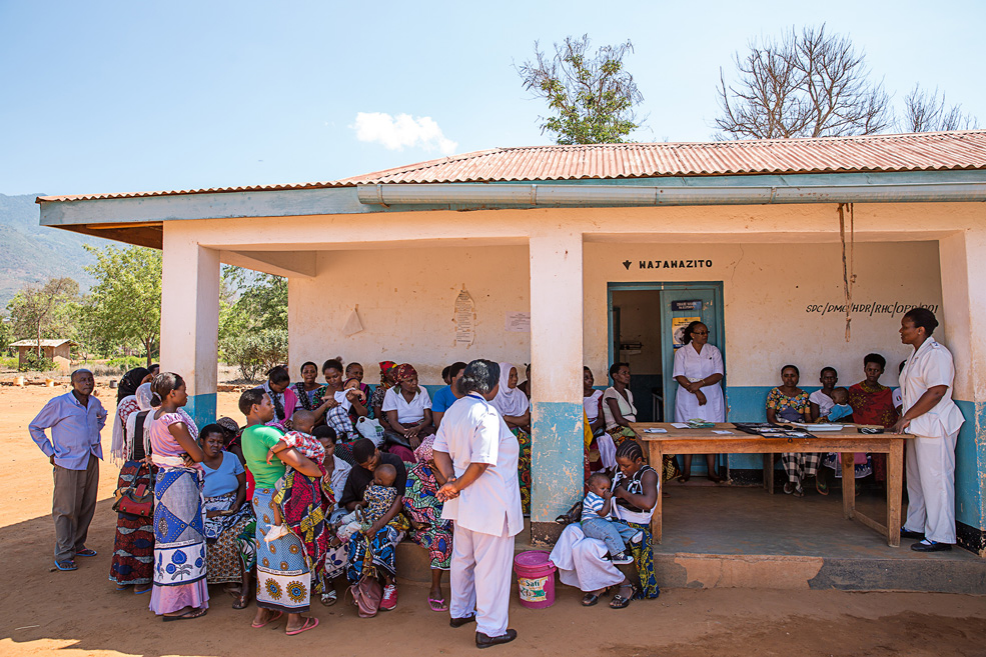
Clients of mobile outreach services in Tanzania listen to a group family planning counseling session. © 2015 Sala Lewis/EngenderHealth

Special family planning days and mobile outreach events served higher client loads than routine services in all 3 countries.

With heightened awareness of FFIC and data showing significant increases in the number of hormonal implant adopters, especially in the DRC, government officials and program managers moved to increase their monitoring of FFIC, especially during high-volume mobile service delivery and special family planning day events. Steps to assess FFIC included improving supervision of counseling and conducting this study of client perceptions of quality and choice.^28^

This study investigated and compared aspects of FFIC and quality service delivery from the client perspective for routine static services, mobile outreach, and special family planning days. Our primary interest was to understand clients' experiences of FFIC and whether elements of FFIC varied by service delivery mode in project catchment areas. We also analyzed characteristics of the family planning clients to understand the populations reached by the 3 service delivery modalities in each country. This article adds to the literature because previous studies have focused their research on FFIC and client satisfaction in private versus public clinics,^29^ the quality of mobile outreach services,^3^ and how client-centered counseling can improve client satisfaction.^30^^,^^31^ While many tools exist to aid programs in human-rights based programming and monitoring, they have seldom been brought to scale in national programs or measured systematically.^32^ This study goes beyond the current literature by comparing FFIC and client satisfaction among 3 service delivery modalities and underscoring the value of using existing tools to provide valuable monitoring data to inform program needs.

## METHODS

We conducted a cross-sectional, facility-based survey between April and July 2015 at 30 EngenderHealth supported-facilities: 9 in the DRC, 13 in Tanzania, and 8 in Uganda. In Tanzania and Uganda, the facilities selected were in peri-urban and rural areas, whereas in the DRC, the facilities were in peri-urban and urban areas of Kinshasa. The study purposefully selected facilities geographically spread across districts receiving support from EngenderHealth. Each data collection site had mobile, static, or in the DRC only, special family planning day services during the project period. Facilities were chosen for data collection by service delivery mode, based on the timing of family planning service activities, type of support from EngenderHealth (static and special family planning days vs mobile outreach), and expected client load. Some facilities had routine client loads too low to make reaching the desired sample feasible and, thus, were excluded. Facilities for mobile outreach collection were selected based on the scheduled mobile outreach activities during the data collection period.

Trained data collectors conducted exit interviews with clients immediately following their family planning visits. Clients were systematically sampled using an interval based on expected client load and were interviewed privately using a structured questionnaire. Signed informed consent was obtained in local languages prior to the interview. Eligible respondents were women aged 15 to 49 years seeking family planning services during static family planning service delivery or mobile outreach services (or in the case of the DRC only, during special family planning days), and who were not pregnant. Clients aged 15 to 17 years were eligible if they were legally emancipated. All data collectors were women to reduce respondent discomfort with questions related to reproductive behaviors and intentions.

### Outcomes

The main outcomes assessed were measures of FFIC and client satisfaction.

Outcome indicators are defined in detail in Table 1. Indicators 1 through 13 directly correspond to responses to questions from client exit interviews that asked clients to report on the elements of counseling they received and their personal choice in method adoption. The majority of questions required a yes/no response: 1 recorded for a “yes” answer and 0 for “no.” Indicators 1 and 2 were assessed among all respondents, indicators 3 through 9 among all respondents receiving a method, and indicators 3 through 13 among women who received a LARC. The proportion of positive responses were tabulated within the denominator category specified in Table 1.

**TABLE 1.** Indicators of Full, Free, and Informed Choice and Rationale for Inclusion

Indicator	Rationale	Denominator
1. Received an FP method	Clients receiving FP is key to FFIC; however, a client not receiving a method does not necessarily demonstrate a lack of choice. A client may come for removal, other services, or choose not to adopt a method.	All women
2. Reported being asked about reproductive intentions (when or whether a client wants more children)	Provider's knowledge of a client's desire to delay, space, or limit childbearing is important for recommending appropriate methods.	
3. Reported discussing 3 or more methods with provider	Clients should be aware that they have options to select the method best suited for them.	
4. Client given a chance to ask questions	Clients in any clinical setting should be given an opportunity to ask questions.	
5. Obtained FP method of choice	Full choice and free choice are contingent on the client receiving her desired method. The reasons for not receiving the desired method include unavailability of the method, lack of a trained provider, cost, medical contraindication, or other.	Women who adopted an FP method
6. Participated in FP decision making (client chose method by herself, jointly with the provider, or jointly with a partner)	Clients should have agency in choosing their method, either by themselves or together with the provider, with a partner, or with someone else. If the client reported that someone else made the decision for her, a lack of FFIC is indicated.	
7. Counseled on method received	The client being given general information on the method received is key to being informed.	
8. Counseled on benefits of method received	The client being told the benefits (e.g., effectiveness, health benefits) of the method received is key to being informed.	
9. Counseled on side effects of method received	The client being told and understanding the side effects of the method received is key to being informed and can also prevent early discontinuation.	
10. Told where to get implant/IUD removed	A client should know the effort required to have the LARC removed before she adopts it (e.g., long distance travel).	Women who adopted a LARC
11. Told when to get implant/IUD removed	A client should know when to have the LARC removed. This is key to correct use and fulfilling reproductive intentions.	
12. Told could have implant/IUD removed whenever she wanted	A client should know that she is free to discontinue use when desired. This is key to free choice.	
13. Could correctly state the maximum duration of implant/IUD use	This indicator verified that clients understood the maximum duration of use.	
**FFIC composite**: Percentage of FP adopters who responded positively to indicators 3 through 9	These 7 indicators represent the minimum threshold for a client to fully exercise FFIC. All 7 indicators had to have a positive response for this indicator to be satisfied.	Women who adopted an FP method
**FFIC score:** Average number of indicators 3 through 9 for which the response was positive (maximum score of 7)	The average provides a more nuanced view of the differences among service-delivery approaches.	
**FFIC LARC composite:** Percentage of LARC adopters who responded positively to indicators 3 through 13	These 11 indicators represent the minimum threshold for a client to fully exercise FFIC when obtaining a LARC: all regular indicators of FFIC plus 4 specific to LARC. All 11 indicators had to have a positive response for this indicator to be satisfied.	Women who adopted a LARC
**FFIC LARC score**: Average number of indicators 3 through 13 for which the response was positive (maximum score of 11)	The average score provides a more nuanced view of the differences among service delivery approaches.	

Abbreviations: FFIC, full, free, and informed choice; FP, family planning; IUD, intrauterine device; LARC, long-acting reversible contraception.

To examine counseling and choice elements as a whole, not just individually, composite FFIC scores were constructed by summing the positive responses for indicators 3 through 9 for women receiving any method and summing positive responses from indicators 3 through 13 for women receiving a LARC method. FFIC scores were examined in 2 ways: (1) as a proportion of women who reported affirmatively to all elements in that category of user and (2) as a mean score of positive responses. The highest score possible for women receiving any method was 7 and the highest score for a woman receiving a LARC was 11 (Table 1).

Answers from 13 indicators were used to arrive at a final composite FFIC score to measure clients' positive perceptions of and satisfaction with their service delivery experience.

We assessed satisfaction using a 4-point Likert scale: respondent was very dissatisfied, somewhat dissatisfied, somewhat satisfied, or very satisfied. Recognizing that courtesy bias contributes to high levels of satisfaction and that reporting “somewhat satisfied” versus “very satisfied” may indicate a small amount of dissatisfaction, we analyzed elements of satisfaction using a dichotomous variable categorized as “very satisfied” versus the rest.

Client characteristics assessed were reproductive intentions (i.e., desire to space or limit pregnancies), parity, marital status, age, education attained, and literacy as well as 2 proxy measures of socioeconomic status (SES): mobile phone ownership and home electricity. We also examined previous contraceptive use, method preference, and reproductive intentions as well as method received on the day of service.

### Sample Size

The sample size was estimated using client satisfaction as the key outcome of interest. Although satisfaction is not an ideal measure because of the potential for courtesy bias and subjectivity as an indicator of perceived quality of service delivery,^14^^,^^16^^,^^17^ it was one of the only measures we could estimate in advance with relative confidence to calculate sample size. Assuming that approximately 95% of clients would be somewhat or very satisfied, a sample of 73 was adequate at the 95% confidence level to assess client satisfaction at the service modality level. Historical service statistics helped determine an appropriate sampling interval by facility and service delivery modality in order to reach desired sample size using systematic sampling, with a minimum of 73 per service delivery modality. The desired sample was then divided across facilities participating in project-supported mobile outreach or special family planning days during the data collection period.

### Data Analysis

Trained data clerks entered data using the Census and Survey Processing System (U.S. Census Bureau and ICF International, Washington DC, USA) data processing software package and cleaned and analyzed the data using Stata version 13 (StataCorp, College Station, TX, USA). We summarized client characteristics using means or medians for continuous variables and proportions for dichotomous variables. We also compared the characteristics of mobile outreach service attendees (and in the case of the DRC, special family planning days) against static service clients through 1-way measures of association: *t* tests for continuous variables, such as age, and chi-square tests for categorical outcomes. To assess the association between each of the primary outcomes—1 through 13 in Table 1, the FFIC composite statistics, and client satisfaction—with mode of service delivery (static or non-static) we used logistic regression. Mode of service delivery was the only predictive variable included in the models. We did not adjust for client characteristics, because client characteristics, such as age, marital status, education, and socioeconomic status, should not affect counseling practices or FFIC. Mean FFIC scores were compared using ANOVA instead of logistic regression. In the DRC, special family planning days and mobile outreach were combined into a single group because they are both non-static, high-volume modes of service delivery supported with many of the same program inputs and have similar client profiles for those attending events. Standard errors for each estimate presented were adjusted for by clustering by facility, the primary sampling unit. Only adjusted estimates are reported; all *P* values reported are 2-sided; and differences in statistical significance at the *P*≤.05, *P*≤.01, and *P*≤.001 were noted.

### Ethical Approvals

The research protocol and materials were reviewed and approved by the Western International Review Board in the United States, the National Institute for Medical Research in Tanzania, the Research Ethics Committee of Makerere University in Uganda, and the Ethical Committee of the Public Health School of Kinshasa in the DRC.

## RESULTS

A total of 614 women were screened for inclusion, of whom 596 were eligible; 587 consented to be interviewed; and 585 women completed the interview. Data were collected from 150 respondents in Uganda (90 static; 60 mobile outreach); 200 respondents in Tanzania (100 static; 100 mobile outreach); and 235 respondents in the DRC (55 static; 120 mobile outreach; 60 special family planning days). A smaller than anticipated client flow at static services in the DRC posed challenges for data collection, and timing and budgetary constraints resulted in a smaller than planned sample in family planning days in the DRC and mobile outreach in Uganda.

### Client Characteristics

Client characteristics varied by mode of service delivery in the DRC. Overall, women seeking family planning at mobile outreach and special family planning days were similar to each other; however, compared with clients attending static services, they had less education, money, and history of family planning use and were more likely to have a preexisting preference for implants. Women at mobile outreach and special family planning days were less literate compared with women at static services (60.8%, *P*≤.01, and 63.3%, *P*≤.05, respectively, compared with 83.3%), and less likely to own a mobile phone (46.7%, *P*≤.001, and 56.7%, *P*≤.05, respectively, compared with 74.6%) (Table 2). In terms of contraceptive history, women attending mobile outreach services and special family planning days had similar levels of modern method use, which were significantly lower than women attending static services (66.7% and 65.0%, respectively, compared with 81.8%, *P*≤.05). For both mobile outreach and special family planning days, clients preferred LARCs/permanent methods, and implants in particular, with 86.7% at mobile outreach services (*P*≤.001) and 85.0% at special family planning days (*P*≤.001) favoring the implant, compared with 58.2% of clients at static services. The only significant differences between clients attending mobile outreach services and clients at special family planning days were that the women attending special family planning days were older (28.9 years compared with 26.9 years, *P*≤.05) and less likely to have electricity than mobile outreach clients (63.3% compared with 82.5%, *P*≤.01).

**TABLE 2.** Profile of Family Planning Users by Country and Service Delivery Modality

	DRC (N=235)^a^			Tanzania (N=200)		Uganda (N=150)	
	Static n=55	Outreach n=120	FP Day n=60	Static n=100	Outreach n=100	Static n=90	Outreach n=60
**Age**							
Age, years, mean	29.2	26.9*^b^	28.9*^c^	27.6	28.8	26.9	27.9
Age, years, range	19–49	17–45	18–44	18–49	17–46	17–47	17–41
Age groups, years, %							
15–19	1.8	11.%	5.0	5.0	11.0	11.1	5.0
20–24	25.5	30.0	18.3	41.0	26.0	28.9	23.2
25–29	29.1	22.5	35.0	17.0	19.0	26.7	33.3
30–34	16.4	24.2	23.3	19.0	13.0	16.7	23.3
35–39	23.6	9.2	10.0	8.0	20.0	14.4	11.7
≥40	3.6	2.5	8.3	10.0	11.0	2.2	3.3
**Marital Status**							
Married or in union, %	81.8	68.3	81.7	84.0	82.0	80.0	93.3*
**No. of Children**							
No. of children, mean	3.8	4.1	4.1	2.8	3.4	3.3	4.1*
No. of children, range	1–10	0–10	1–9	0–11	0–11	0–9	0–11
No. of children, distribution, %							
0–3	49.1	44.2	38.3	76.0	62.0*	54.3	43.3
>3	50.9	55.8	61.7	24.0	38.0*	45.6	56.7
**Education**							
Received at least some secondary education, %	83.6	75.0	75.0	25.0%	15.0	31.1	25.0
Read some/all sample sentence,^e^ %	83.3	60.8**^b^	63.3*^d^	85.0	83.0	80.0	75.0
**Socioeconomic Status**							
Owns a mobile phone, %	74.6	46.7***^b^	56.7*^d^	59.0	38.0**	63.3	58.3
Has electricity, %	87.3	82.5	63.3**^c,d^	26.0	24.0	30.0	25.0
**Occupation** ^f^							
Housewife/not working, %	27.3	40.8	41.7	16.0	12.0	18.9	13.3
Farmer, %	1.8	2.5	3.3	59.0	82.0***	55.6	65.0
Trader/business owner, %	47.3	43.3	43.3	15.0	2.0***	20.0	15.0
**Contraceptive History**							
Ever used modern FP, %	81.8	66.7*^b^	65.0*^d^	91.0	90.0	98.9	100.0
Ever used non-condom modern FP, %	74.6	46.7***^b^	51.7*^d^	83.0	84.0	88.9	96.7
Ever used LA/PM, %	5.5	2.5	3.3	33.0	36.0	25.6	15.0
**Method Preferences**							
Had a preference for implant, %	58.2	86.7***^b^	85.0***^d^	41.0	55.0	25.6	51.7***
Had a preference for LA/PM, %	58.2	86.7***^b^	88.3***^d^	47.0	62.0*	28.9	53.3**
Had a preference for short-acting method, %	29.1	2.5***^b^	6.7**^d^	37.0	20.0**	63.3	33.3***
**Fertility Desires**							
Wants no more children, %	34.6	41.7	30.0	14.0	32.0**	25.6	41.7*
Wants child 2 or more years, %	49.1	40.0	50.0	67.0	55.0	44.4	38.3
Doesn't know when or if want more, %	9.1	10.0	16.7	14.0	3.0	5.6	6.7

Abbreviations: DRC, Democratic Republic of the Congo; FP, family planning; LA/PM, long-acting or permanent method.

a One-way analyses of statistical significance were conducted between mobile outreach and static services; special family planning days and static services; and special family planning days and mobile outreach.

b Difference between mobile outreach and static services was statistically significant.

c Difference between special family planning days and mobile outreach was statistically significant.

d Difference between special family planning days and static services was statistically significant.

e Women who were visually impaired or who did not read the language on the card (n=5) were excluded.

f Only the 3 most common occupations overall are listed, so categories do not add up to 100%.

* *P*≤.05;

** *P*≤.01;

*** *P*≤.001

For both mobile outreach and special family planning days, clients preferred LARCs/permanent methods, especially implants, compared with clients at static clinics.

In Tanzania, women attending mobile outreach were more likely to have 3 or more children (38.0% compared with 24.0%, *P*≤.05), were less likely to own a mobile phone (38.0% compared with 59.0%, *P*≤.01), and were more likely to be farmers (82.0% compared with 59.0%, *P*≤.001) than women at static services (Table 2). Although preference for an implant was not as pronounced as in the DRC, there was a clear preference for LARCs/permanent methods among mobile outreach clients compared with static service clients (62.0% compared with 47.0%, *P*≤.05). The women's reproductive intentions also varied, with 32.0% attending mobile outreach indicating that they wanted no more children compared with 14.0% at static services (*P*≤.01).

There were fewer differences between users of static and mobile outreach services in Uganda than in Tanzania and the DRC; however, differences did emerge. Women attending mobile outreach were more likely to be married or in union (93.3% compared with 80.0%, *P*≤.05), had a higher mean number of children (4.1 compared with 3.3, *P*≤.05), had a stronger preference for implants (51.7% compared with 25.6%, *P*≤.001), and had a greater desire to have no more children (41.7% compared with 25.6%, *P*≤.05) compared with women attending static services (Table 2).

### Method Adoption

In all countries, women's preference for LARC/permanent methods, and especially for implants, was reflected in the method adopted (Table 3). Significantly higher percentages of women attending non-static services in the 3 countries adopted an implant and significantly lower percentages adopted an injectable compared with women at static services. Few clients in any modality adopted IUDs, condoms, or pills, although all of these methods were available at all service modalities. In the DRC, 63.4% of women attending static services adopted implants and 29.1% adopted injectables. In contrast, 94.2% of women attending mobile outreach and 96.7% at family planning day events adopted implants (both *P*≤.001), whereas only 3.3% and 1.7% adopted injectables, respectively (both *P*≤.001). In Tanzania, 64.0% of women attending mobile outreach adopted an implant compared with 46.0% at static services (*P*≤.01), and only 8.0% attending mobile outreach adopted an injectable compared with 24.0% at static services (*P*≤.01). Finally, in Uganda, 43.3% of women attending mobile outreach adopted an implant and 23.3% adopted an injectable compared with 24.4% adopting an implant (*P*≤.05) and 51.1% adopting an injectable (*P*≤.01) at static sites. The implant was the method most often adopted in each country and at each service delivery modality, with the exception of Uganda, where slightly more than half (51.1%) of the clients attending static services chose an injectable.

**Figure:**
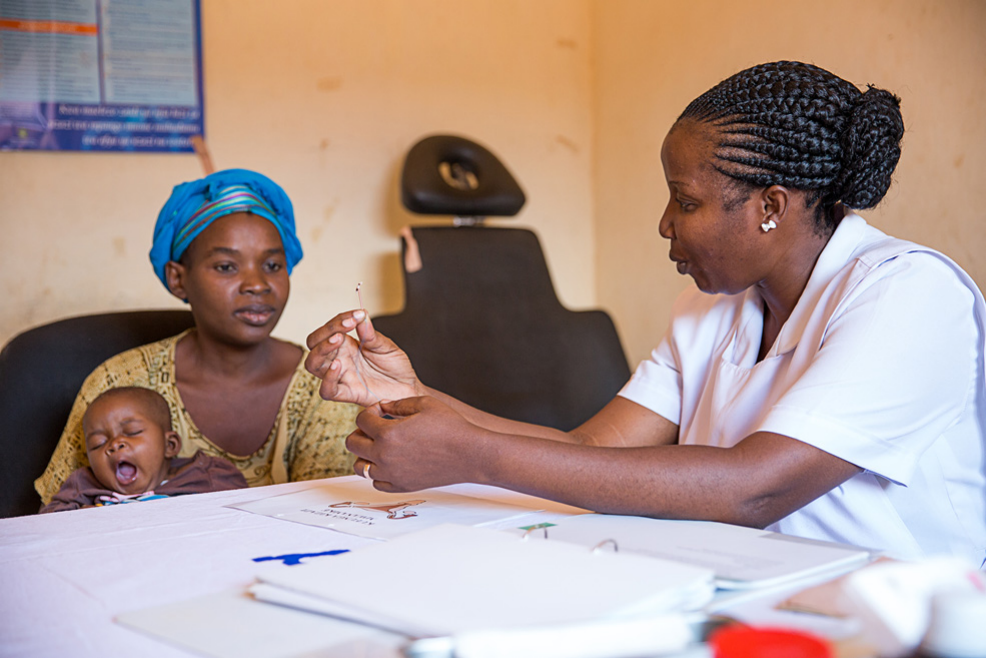
A family planning provider in Tanzania counsels a client of mobile outreach services on IUDs. © 2015 Sala Lewis/EngenderHealth

**TABLE 3.** Primary Family Planning Method Received, by Country and Service Delivery Approach

Method	DRC (N=235)^a^			Tanzania (N=200)		Uganda (N=150)	
	Static (%) n=55	Outreach (%) n=120	FP Day (%) n=60	Static (%) n=100	Outreach (%) n=100	Static (%) n=90	Outreach (%) n=60
No method	5.5	2.5	0.0	6.0	9.0	8.9	18.3
Condom	0.0	0.0	0.0	3.0	1.0	2.2	0.0
Pill	1.8	0.0	0.0	13.0	9.0	11.1	11.7
Injectable	29.1	3.3***^b^	1.7***^c^	24.0	8.0**	51.1	23.3**
Implant	63.4	94.2***^b^	96.7***^c^	46.0	64.0**	24.4	43.3*
IUD	0.0	0.0	1.7	8.0	4.0	2.2	3.3
Tubal ligation	0.0	0.0	0.0	0.0	5.0*	0.0	0.0

Abbreviations: DRC, Democratic Republic of the Congo; FP, family planning; IUD, intrauterine device.

* *P*≤.05;

** *P*≤.01;

*** *P*≤.001.

a One-way analyses of statistical significance were conducted between mobile outreach and static services, special family planning days and static services, and family planning days and mobile outreach.

b Difference between mobile outreach and static services was statistically significant.

c Difference between special family planning days and static services was statistically significant.

### Composite and Individual Measures of FFIC

Several significant differences were found in elements of FFIC between service delivery modalities in each country. The FFIC composite indicator for all clients adopting family planning showed that fewer than half the clients in any country or for any service delivery modality reported experiencing all aspects of FFIC (range, 19.2% to 48.1%) (Table 4). Differences between service delivery modalities were significant only in Tanzania, where 31.9% of clients attending mobile outreach services reported experiencing all elements of FFIC compared with 48.9% of clients of static services (odds ratio [OR]=0.5; 95% confidence interval [CI], 0.4 to 0.7; *P*≤.001). The trend was the same in the DRC, although the results were not significant. In all 3 countries, the average number of FFIC elements for which the response was positive (FFIC mean score) was between 4.8 and 6.1 (of 7). In Tanzania, women in static services reported experiencing on average 6.1 elements of FFIC compared with 5.6 in mobile outreach (*P*=.02); no other significant differences were found. When looking exclusively at LARC adopters, differences between service modalities were significant only in Tanzania: 26.4% of clients of mobile outreach services reported experiencing all aspects of FFIC, including additional questions related to LARC, compared with 40.7% of clients of static services (OR=0.5; 95% CI, 0.3 to 0.8; *P*≤.01). The directionality of differences in the DRC and Uganda was the same, but again, was not significant. The average number of FFIC elements, including LARC elements, for which the response was positive was between 8.3 and 9.8 (of 11) for all countries. The difference was again only significant in Tanzania (9.8 in static services, 8.7 in mobile outreach, *P*≤.01). Importantly, between 92.7% and 100% of clients who received a family planning method obtained their method of choice in all countries and modalities, and the large majority of clients (74.0% to 97.9%) also reported that they made the decision to use family planning either by themselves or jointly with their partner or provider.

**TABLE 4.** Measures of the Association of Service Delivery Approach With Elements of FFIC, by Country

Outcome	DRC (N=235)			Tanzania (N=200)			Uganda (N=150)		
	Static (n=55) %	Outreach/Special FP Day (n=180) %	OR (95% CI)^a^ or *P* Value^b^	Static (n=100) %	Outreach (n=100) %	OR (95% CI)^a^	Static (n=90) %	Outreach (n=60) %	OR (95% CI)^a^or *P* Value^b^
**All women**									
1. Obtained a method	94.6	98.3	3.4 (0.4,27.9)	94.0	91.0	0.6 (0.1,5.9)	91.1	81.7	0.4 (0.1,2.3)
2. Reported being asked about reproductive intentions	80.0	59.4	0.4 (0.1,2.7)	94.0	75.0	0.2 (0.1,0.7)**	64.4	55.0	0.7 (0.4,1.3)
3. Reported discussing three or more methods with provider	65.5	78.3	1.9 (0.4,10.1)	90.0	81.0	0.5 (0.2,1.1)	26.7	41.7	2.0 (0.3,12.5)
4. Given a chance to ask questions	61.8	48.3	0.6 (0.1,2.4)	72.0	70.0	0.9 (0.7,1.2)	75.6	96.7	9.4 (0.8,115.2)
**Women who adopted an FP method**	**n=52**	**n=177**		**n=94**	**n=91**		**n=82**	**n=49**	
5. Obtained FP method of choice	100.0	98.3	NA	92.6	96.7	2.4 (0.2,24.7)	97.6	95.9	0.6 (0.1,2.6)
6. Participated in FP decision making	96.2	74.0	0.1 (0.0,0.4)***	97.9	87.9	0.2 (0.0,2.3)	92.7	89.8	0.7 (0.4,1.4)
7. Counseled on method received	80.8	83.6	1.2 (0.2,8.7)	93.6	87.9	0.5 (0.4,0.6)***	39.0	42.9	1.2 (0.2,6.1)
8. Counseled on benefits of method received	69.2	75.1	1.4 (0.3,6.6)	84.0	78.0	0.7 (0.4,1.1)	76.8	85.7	1.8 (0.6,5.4)
9. Counseled on side effects of method received	69.2	62.7	0.7 (0.2,3.2)	75.5	58.2	0.5 (0.2,1.1)	63.4	65.3	1.1 (0.6,1.9)
**FFIC composite:** Percentage of women who adopted an FP method responding positively to ALL indicators 3 through 9	48.1	19.2	0.3 (0.1,1.3)	48.9	31.9	0.5 (0.4,0.7)***	22.0	20.4	0.9 (0.1,5.6)
**Women who adopted a LARC**	**n=35**	**n=172**		**n=54**	**n=68**		**n=24**	**n=28**	
10. Told where to get implant/IUD removed	74.3	70.4	0.8 (0.2,3.8)	96.2	79.1	0.1 (0.1,0.2)***	79.2	92.9	3.4 (1.4,10.2)*
11. Told when to get implant/IUD removed	85.7	81.4	0.7 (0.1,4.3)	98.1	89.4	0.2 (0.0,2.6)	91.7	96.4	2.5 (1.4,4.2)***
12. Told could have implant/IUD removed whenever wanted	77.1	76.7	1.0 (0.1,6.7)	84.9	68.7	0.4 (0.1,1.3)	83.3	96.4	5.4 (0.6,51.6)
13. Could correctly state when implant or IUD would expire	85.7	80.8	0.7 (0.1,4.1)	88.9	86.8	0.8 (0.5,1.4)	91.7	92.9	1.2 (0.6,2.2)
**FFIC LARC composite:** Percentage of women who adopted a LARC responding positively to ALL indicators 3 through 13	37.1	15.7	0.3 (0.1,1.8)	40.7	26.4	0.5 (0.3,0.8)**	20.8	17.9	0.8 (0.1,5.1)
**FFIC mean score**									
FFIC mean score: Average number of indicators 3 through 9 for which response was positive (highest possible score = 7) among women who adopted an FP method	5.5	5.2	.23	6.1	5.6	.02*	4.8	5.1	.15
FFIC mean LARC score: Average number of indicators 3 through 13 for which response was positive (highest possible score = 11) among women who adopted a LARC	8.5	8.3	.70	9.8	8.7	.002**	8.3	8.6	.33

Abbreviations: CI, confidence interval; DRC, Democratic Republic of the Congo; FFIC, full, free, and informed choice; FP, family planning; IUD, intrauterine device; LARC, long-acting reversible contraception; OR, odds ratio.

* *P*≤.05;

** *P*≤.01;

*** *P*≤.001.

a Error estimates are adjusted for clustering by facility.

b *P* values are reported for the FFIC mean scores at the end of the table.

Most family planning clients obtained their method of choice in all countries and service modalities.

In the DRC, only 1 individual measure of FFIC—the client participating in family planning decision making—showed a significant difference between the service delivery approaches: 74.0% of clients of mobile outreach and special family planning days reported such joint decision making compared with 96.2% of clients of static services (OR=0.1; 95% CI, 0.0 to 0.4; *P*≤.001) (Table 4). No other individual measures of FFIC were significant, and there was no clear trend in directionality among the indicators. However, there was a notable difference in the percentage of women who said that they were given a chance to ask questions: less than half (48.3%) attending mobile outreach/special family planning days compared with 61.8% at static services. It should also be noted that although differences in the FFIC composite indicator were not significant, only 1 in 5 clients attending mobile outreach or special family planning days reported experiencing all measures of FFIC, compared with 1 in 2 clients at static services.

Similar to the DRC, most of the indicators of FFIC did not differ significantly by service delivery modality in Tanzania. The overall trend suggested that FFIC was better at static services compared with mobile outreach, with 3 individual indicators significantly so: reporting that the provider asked about their reproductive intentions (OR=0.2; 95% CI, 0.1 to 0.7; *P*≤.01), reporting that they were counseled on the method received (OR=0.5; 95% CI, 0.4 to 0.6; *P*≤.001), and reporting that they were told where to have their implant or IUD (OR=0.1; 95% CI, 0.1 to 0.2; *P*≤.001). Other elements of counseling, such as “counseled on side effects of method received” and “told could have implant/IUD removed whenever wanted” differed between the 2 service delivery modalities but did not reach the level of statistical significance.

In Uganda, only 2 indicators—told where, and when, to get an IUD/implant removed—showed significant differences, both among LARC adopters. Both suggested superior counseling at mobile outreach compared with static services, in contrast to Tanzania (Table 4). Overall, where there were sizable differences between the 2 service delivery modalities on indicators that did not rise to the level of statistical significance, most measures of FFIC were better for mobile outreach services.

We did not make any statistical comparisons among the countries because their family planning programs are in different stages of development, and there were likely differences between populations served. However, we observed that in Tanzania, the absolute value of most indicators of FFIC was over 80% (Table 4). In contrast, many values in Uganda and the DRC were much lower, while variability among different measures was high. In the DRC, measures ranged from 48.3% for being given a chance to ask questions at outreach services/special family planning day events to 100% for obtained method of choice at static services. In Uganda, the measures ranged from 26.7% for provider discussed 3 or more methods at static services to 97.6% for obtained method of choice at static services.

### Satisfaction

Although overall satisfaction did not differ statistically by mode of service delivery in the DRC, significantly lower percentages of respondents attending outreach services/special family planning days than those at static services reported being “very satisfied” for 5 of the 8 individual measures of satisfaction: amount of time waited, amount of family planning information given, the opportunity to ask questions, the way the client was treated by staff, and the way she was treated by the provider (Table 5). For the remaining measures, the trend was the same, although not statistically significant.

**TABLE 5.** Proportion of Clients Reporting Being “Very Satisfied” With Aspects of Services, by Country and Service Delivery Modality

	DRC (N=235)			Tanzania (N=200)			Uganda (N=150)		
	Static (n=55) %	Outreach/SpecialFP Day (n=180) %	OR (95% CI)^a^	Static (n=100) %	Outreach (n=100) %	OR (95% CI)^a^	Static (n=90) %	Outreach (n=60) %	OR (95% CI)^a^
Amount of time waited to see a provider	69.1	32.2	0.2 (0.1, 0.8)*	94.0	84.0	0.3 (0.1, 0.8)*	27.8	66.7	5.2 (2.3, 12.0)***
Privacy of your consultation with the provider	78.2	42.2	0.2 (0.0, 1.1)	94.0	91.0	0.6 (0.0, 0.8)***	72.2	95.0	7.3 (1.6, 33.3)*
The cleanliness of the facility	65.5	25.0	0.2 (0.0, 1.2)	85.0	81.0	0.8 (0.2, 2.4)	41.1	61.7	2.3 (0.7, 8.0)
The amount of FP information you were given	65.5	27.8	0.2 (0.0, 0.8)*	88.0	85.0	0.8 (0.2, 2.6)	56.7	73.3	2.1 (1.2, 3.7)**
The opportunity to ask questions	45.5	16.9	0.2 (0.1, 0.7)**	87.0	80.0	0.6 (0.2, 1.7)	71.1	95.0	7.7 (1.9, 31.0)**
The quality of the FP counseling you received	61.1	33.9	0.3 (0.1, 1.3)	91.0	85.0	0.6 (0.3, 1.1)	46.7	68.3	2.5 (1.4, 4.3)**
The way you were treated by staff	87.3	43.3	0.1 (0.0, 0.4)***	95.0	88.0	0.4 (0.2, 0.7)**	67.8	86.7	3.1 (1.2, 8.3)*
The way you were treated by the provider	89.1	43.3	0.1 (0.0, 0.7)*	95.0	88.0	0.4 (0.1, 1.5)	87.8	98.3	8.2 (0.7, 88.9)
Overall satisfaction with services	79.6	40.6	0.2 (0.0, 2.0)	96.0	89.0	0.3 (0.2, 0.6)	78.9	91.7	2.9 (0.7, 11.8)

Abbreviations: CI, confidence interval; DRC, Democratic Republic of the Congo; FP, family planning; OR, odds ratio.

* *P*≤.05;

** *P*≤.01;

*** *P*≤.001.

a Error estimates are adjusted for clustering by facility.

In Tanzania, lower percentages of mobile outreach clients than static service clients also reported satisfaction with aspects of services, with 3 measures significantly so: amount of time waited, privacy of consultation, and the way the client was treated by staff (Table 5).

Although overall satisfaction did not differ statistically by mode of service delivery in Uganda, significantly higher percentages of respondents attending outreach services/special family planning days than those at static services reported being “very satisfied” on 6 of the 8 individual measures of satisfaction: amount of time waited, privacy of consultation, amount of family planning information given, opportunity to ask questions, and quality of family planning counseling received, and the way the client was treated by staff (Table 5).

As stated earlier, statistical comparisons were not made among the countries. Nevertheless, it is notable that the proportion of clients reporting being very satisfied in the DRC varied widely, from just 16.9% of women attending mobile outreach services/special family planning days for the opportunity to ask questions and 25.0% for facility cleanliness to 89.1% at static services for treatment by the provider (Table 5). In Uganda, there was also pronounced variation, with just over a quarter (27.8%) of clients at static services being very satisfied with the amount of time they waited to nearly all (98.3%) clients at mobile outreach services being very satisfied with the way they were treated by the provider. In contrast, at least 80% of clients reported being very satisfied on every measure in Tanzania.

## DISCUSSION

The analysis of the composite FFIC indicator suggests that, overall, clients experienced greater FFIC at static services compared with mobile outreach in Tanzania, while significant differences were not found in the DRC or Uganda. Although fewer than half of clients reported experiencing all aspects of FFIC in all countries and for all modalities, the FFIC mean score indicates that clients—all family planning adopters and LARC-only adopters—experienced the majority of elements of FFIC. The fact that few of the individual indicators of FFIC were significant in any country but showed greater differences when examined as a composite indicator may be an issue of power. It is, therefore, important to look at trends in the domains of FFIC as well as the composite measure and mean score.

The results indicate that women were equally likely to obtain a family planning method and, specifically, the method they wanted at all service delivery modalities in each country. It is important to note that a higher percentage of women who came to non-static service sites had a preexisting preference for a LARC than those attending static services in all 3 countries. Women coming to non-static services may have sought those services specifically because they knew these methods would be available, thereby potentially masking a difference in method availability between service delivery modalities. The women's preference for LARCs at mobile outreach and special family planning days suggests that the high levels of implant uptake at these services were likely related primarily to preexisting preferences, rather than the unavailability of other methods or provider bias. In particular, in the DRC, the fact that methods were free during special family planning days and mobile outreach may have attracted clients who were waiting specifically for free events in order to obtain LARCs, which are normally costly.

Findings for the other individual indicators of FFIC were mixed. One indicator showed better performance at static services in the DRC; 3 indicators showed better performance at static services in Tanzania; and 2 indicators showed better performance at mobile outreach in Uganda. In Tanzania, the better performance of static services may be a product of the lower volume of clients compared with mobile outreach, resulting in longer counseling sessions. However, we did not measure the length of counseling sessions for each client and, therefore, cannot be certain of this interpretation. In Uganda, the indicators with better results for mobile outreach services were all related to LARCs, possibly reflecting that providers who routinely participate in mobile outreach events are more skilled in the provision of and counseling for LARCs. However, the mobile outreach model in Tanzania and Uganda was similar; therefore, one would expect similar outcomes. It is also plausible that when a client arrives having already decided on a method—which was more likely for non-static services in all countries—providers are less likely to give full counseling, assuming—perhaps incorrectly—that the client has all the information she needs. In the DRC, there was no clear trend, indicating that clients at all modalities were equally likely to experience aspects of FFIC. Facilities that provided static services received more routine support and supervision than facilities that held mobile outreach, which may have affected outcomes as well, though the pattern of impact is not clear.

Questions on counseling specifically asked whether “the provider” counseled the client about a particular element. Group counseling is common at mobile outreach services and special family planning day events. It is possible that some clients responded “no” to some elements of counseling because they received information from a different staff member, not the provider who gave her the method. This may have contributed to the low number of clients at both service delivery modalities in Uganda who stated that the provider discussed 3 or more methods with them and that the provider counseled them on the method they received. It is common for a provider to review the array of methods available during group counseling, whereas the accepted method may be given by a different provider. A more nuanced questionnaire could inform this understanding of the data.

Clients attending mobile outreach and special days tended to be of lower SES and education level. It is possible that women of lower SES might not understand all of the information during counseling, which, in turn, may have affected their reporting of FFIC. In practice, it is not possible to separate the service delivery modality from the profile of client reached. It is therefore important to consider how the client profile may affect FFIC in addition to the service delivery modality.

It is essential to note that even when differences between service delivery modalities were not found, some of the indicators of FFIC should be improved in all countries and for all modalities. For example, at least one-quarter of clients in each country and for each service delivery modality reported that they were not counseled on potential method side effects. In some cases, this result may have been because a client was a continuing user of a method; however, it is important to ensure that all clients are given accurate information about their method options and the benefits and risks of family planning. This is particularly true when examining whether clients were given a chance to ask questions. Although there were no significant differences between modalities in any country, Uganda was the only country where more than three-quarters of clients at any modality reported being able to ask questions. All clients should have the opportunity to ask questions. Despite this, nearly all clients received their method of choice, the large majority of clients reported independent or joint decision making, and there was no indication that any client rights were violated. More research is needed to understand why clients may not be experiencing all aspects of FFIC and how to better support providers to deliver quality counseling in various service delivery modalities.

Across all modalities, clients were often not counseled on potential side effects or given the opportunity to ask the provider questions.

Indicators of satisfaction appeared to align with indicators of FFIC; that is, in the modality where clients reported superior indicators of FFIC, they were also more likely to be very satisfied with various aspects of services and counseling. Overall, this indicates that ensuring that clients experience FFIC may increase client satisfaction with services, though specific analysis of any such correlation would be needed to investigate this concept. Further, some elements of services that may affect satisfaction, such as wait time, are beyond FFIC and not likely to be correlated. The proportion of women reporting being very satisfied with a variety of individual indices was notably low in both service delivery modalities in the DRC and Uganda, suggesting that improvements could be made to all services.

Although no statistical comparisons were made among countries, the relatively lower experiences of elements of FFIC and of satisfaction in the DRC and Uganda compared with Tanzania is notable. It is not possible to extrapolate from these data why this difference occurred; however, the prevalence of family planning services, the maturity of the family planning programs, the funding and political environment, and other external factors may have affected providers' abilities to deliver quality services. The implementation of the ExpandFP program also differed from country to country, with varying numbers of providers trained in family planning counseling, a different reach of the program, and slightly different models for mobile outreach and special family planning days. These environmental factors may also affect clients' expectations and experiences with family planning services.

The differences seen in client characteristics between women attending mobile outreach or special family planning days compared with those at static services are also important to consider in terms of the populations reached in the 3 countries. Findings indicate that, overall, mobile outreach and special family planning day services reached women of lower SES than static services, thus underscoring the importance of non-static service delivery options in reaching more disadvantaged and vulnerable populations. The lower proportion of women with a history of modern family planning use also indicates that mobile outreach and special family planning days may reach clients with a long-standing unmet need, particularly an unmet need for limiting childbearing. These differences in characteristics indicate that mobile outreach and special family planning days are important strategies for increasing access to family planning. Moreover, the higher comparative uptake of LARC by women attending mobile outreach and special family planning days, and the possibility that many women came to these services with these methods already in mind, indicate that non-static services are important ways to increase access to these underutilized methods for underserved populations. The women's SES may have also affected how providers counseled them and/or how the clients experienced, understood, and recalled that counseling.

Further research is needed to explore the reasons for differences in FFIC and to determine what approaches may effectively ensure that providers enable all clients, especially women of lower SES, to make FFIC. It is equally important to ensure that the various service delivery modes, including those crucial to reaching underserved populations with underutilized methods, expand access to family planning while offering quality counseling and FFIC for all clients. Existing tools that can be used to monitor and improve clients' experiences of FFIC can and should be brought to scale at national levels—across the private, public, and non-profit spheres—and used to continually improve services. Additionally, client–provider interactions can and should be tailored to meet individual client needs: some clients may want more information than others, returning clients may be happy with their method and not need or want counseling on other methods, while others may want to hear about an array of options. Qualitative research on both the client and provider experiences with counseling can help program planners and implementers to better understand these dynamics and how to measure them. Observations of client–provider interactions are also important to understand the reasons why some clients had better experiences with some elements of FFIC than others.

Using various service delivery modes is crucial to reaching underserved populations with underutilized methods and expanding access to family planning.

### Limitations

This research is not generalizable at the country level because the facilities, which were purposively selected in each country, received different levels of project support and, therefore, are not representative of facilities in general. Similarly, public-sector mobile outreach may differ from private-sector or NGO-led outreach, which was not captured here, and the client profile at study facilities may not be representative of clients, in general. Additionally, the study's findings are dependent on client recall of experiences. Recall bias was minimized by interviewing clients immediately following their receipt of services and prior to their exit from the facility. This was a strength of our study design, compared to household surveys, which interview clients about counseling practices long after the event has occurred. Courtesy bias may also have affected the reliability of study results, especially related to measures of satisfaction. This bias was minimized by interviewing clients privately and by non-facility staff conducting the interviews. In addition, measures of the elements of FFIC were limited in our study to the client's perspective. Finally, the relatively small sample size may have limited the power to detect significant differences, in particular, because the desired sample size was not reached in all modalities.

## CONCLUSIONS

The study findings suggest that client experiences of FFIC elements varied among the service delivery modalities, with certain elements scoring lower, and other elements scoring higher, at some non-static service delivery modalities compared with static services. The reasons for this variance may be related to client volume during non-static service delivery events, the profile of clients who attended non-static service delivery, differences in how providers approached women of higher or lower SES, or other unknown factors. In cases where the FFIC scores were lower, provider monitoring, supervision, and follow up on appropriate counseling methods as well as ensuring sufficient staff time for comprehensive counseling may have enhanced clients' satisfaction and experiences of FFIC. Implementers may need to increase staffing, establish a maximum number of clients during special family planning events, or use other approaches to ensure enough time for counseling. Further research is needed to understand the conditions or circumstances that may make FFIC more difficult in mobile outreach settings or during special family planning days. Special family planning days and mobile outreach days play a key part in expanding access to family planning, and the large majority of clients were able to obtain the method of their choice in these events, the majority of whom chose a LARC. Each service delivery modality poses different challenges to providers to provide quality services and counseling, and these must be accounted for in program planning.

Despite some high scores, most elements of FFIC for all service delivery modalities and in all countries still showed room for improvement. Women who adopt family planning should receive high-quality care, including FFIC. This study shows the importance of monitoring FFIC as programs expand access to family planning services and methods. It is not enough to reach clients with methods and services, clients should be empowered to make decisions fully, freely, and with correct and complete information.

## Notes

**En français**

**Perceptions des clients concernant la qualité et le choix au sein des services statiques, mobiles et lors des journées planification familiale dans 3 pays africains**

Dans les 3 pays, quasiment toutes les femmes ont obtenu la méthode de leur choix, sachant que davantage de clientes des services de sensibilisation mobile et des journées spéciales planification familiale avaient auparavant une préférence pour les implants, par rapport aux clientes des services statiques. Les clientes des différents types de service ont déclaré avoir bénéficié de la plupart des éléments du choix total, libre et éclairé, certains aspects peuvent néanmoins être améliorés, comme par exemple les conseils relatifs aux éventuels effets secondaires et le fait de permettre aux clientes de poser des questions.

**RÉSUMÉ**

**Contexte :** L'utilisation de méthodes réversibles de longue durée d'action a considérablement augmenté en République démocratique du Congo (RDC), en Tanzanie et en Ouganda. L'adoption des méthodes réversibles de longue durée d'action est particulièrement élevée pendant les campagnes de sensibilisation mobile et les journées spéciales planification familiale. Il conviendrait donc d'examiner les perceptions des clientes et leur expérience en matière de choix total, libre et éclairé dans différentes modalités de prestation de services.

**Méthodes :** Entre avril et juillet 2015, nous avons conduit une enquête transversale auprès des clientes de planification familiale pour évaluer le choix total, libre et éclairé et la satisfaction des clientes par rapport aux services statiques, mobiles et lors des journées spéciales planification familiale en RDC (n=9 sites), Tanzanie (n=13), et Ouganda (n=8). Cette étude a permis de mener des recherches sur les perceptions des clientes eu égard aux 13 éléments du choix total, libre et éclairé notamment la mesure de la qualité des conseils et de la satisfaction des personnes interviewées à propos des différentes approches en matière de prestation de service. Les résultats composites du choix total, libre et éclairé ont été interprétés et analysés comme correspondant au pourcentage de femmes qui ont répondu par l'affirmative à l'ensemble des éléments et au score moyen de réponses positives. La satisfaction a été évaluée en utilisant une échelle de Likert de 4 points. Nous avons eu recours à la régression logistique pour évaluer le lien entre les résultats primaires et le mode de prestation de service.

**Résultats :** Au total, nous avons interviewé 585 femmes (n=150 en Ouganda, n=200 en Tanzanie, et n=235 en RDC). La grande majorité des clientes de tous les pays et de l'ensemble des modalités ont obtenu la méthode de leur choix. Comparées aux clientes de services statiques les clientes des services mobiles et des journées spéciales planification familiale ont préféré des méthodes réversibles de longue durée d'action et des méthodes permanentes, en particulier les implants. Les mesures composites du choix total, libre et éclairé étaient plus faibles pour la sensibilisation mobile que pour les services statiques en Tanzanie parmi l'ensemble des clientes de planification familiale (coefficient de probabilité [CP]=0,5; *P*≤,001) et parmi celles qui utilisent des méthodes réversibles de longue durée d'action en particulier (CP=0,5; *P*≤,01); aucune différence majeure n'a été constatée en RDC ou en Ouganda. Un score moyen du choix total, libre et éclairé parmi toutes les clientes de services de planification familiale a montré que les clientes de l'ensemble des modalités de tous les pays avaient signalé avoir bénéficié de la plupart des éléments du choix total, libre et éclairé avec des moyennes variant entre 4,8 et 6,1 des 7 éléments. Pour ce qui est des clientes de méthodes réversibles de longue durée d'action en particulier, les scores moyens allaient de 8,3 à 9,8 des 11 éléments. Lorsqu'un pourcentage plus élevé de clientes ont bénéficié d'un degré important de choix total, libre et éclairé, une proportion plus importante de clientes ont également eu tendance à signaler être « très satisfaites » des différents aspects de services et conseils.

**Conclusions :** Les résultats indiquent que les journées spéciales de planification familiale et les services de sensibilisation constituent des moyens importants et viables pour accroître l'accès aux services de planification familiale, notamment aux méthodes réversibles de longue durée d'action, mais il faut accorder une plus grande attention au respect et à la réalisation du choix total, libre et éclairé des clientes dans l'ensemble des modalités de prestation de service.

## Notes

**Peer Reviewed**

**Funding:** Bill & Melinda Gates Foundation.
